# Prevalence of Non-SARS-CoV-2 Respiratory Pathogens and Co-Infection with SARS-CoV-2 in the Early Stage of COVID-19 Epidemic

**DOI:** 10.3390/pathogens11111292

**Published:** 2022-11-04

**Authors:** Huimin Han, Yasin Abdi Saed, Wenzhu Song, Ming Wang, Yafeng Li

**Affiliations:** 1 Department of Nephrology, Shanxi Provincial People’s Hospital (Fifth Hospital) of Shanxi Medical University, Taiyuan 030012, China; 2 Shanxi Provincial Key Laboratory of Kidney Disease, Taiyuan 030012, China; 3 School of Public Health, Shanxi Medical University, Taiyuan 030000, China; 4 Department of Clinical Laboratory, Renmin Hospital of Wuhan University, Wuhan 430060, China; 5 Core Laboratory, Shanxi Provincial People’s Hospital (Fifth Hospital) of Shanxi Medical University, Taiyuan 030012, China; 6 Academy of Microbial Ecology, Shanxi Medical University, Taiyuan 030012, China

**Keywords:** SARS-CoV-2, respiratory pathogen, COVID-19, influenza, co-infection

## Abstract

Background: This study aims to reflect the prevalence of non-SARS-CoV-2 respiratory pathogens and co-infection with SARS-CoV-2 in the early stage of the COVID-19 epidemic, considering SARS-CoV-2 broke out during influenza season and its symptoms resemble those of influenza. Methods: A total of 685 nucleic acid samples of respiratory pathogens were collected from 1 November 2019 to 20 January 2020 and were detected by the 13 Respiratory Pathogen Multiplex Detection Kit and Novel Coronavirus (2019-nCoV) Nucleic Acid Diagnostic Kit. Results: In Wuhan, human rhinovirus was the most frequent infectious pathogen in November (31.5%) and human respiratory syncytial virus appeared the most in December and January (37.1%, 8.6%, respectively). Detection of SARS-CoV-2 first appeared from January 1 to January 10. Generally, 115 patients of 616 patients (18.7%) from Wuhan were infected with SARS-CoV-2, and only two children were co-infected with other respiratory pathogens. In Taiyuan, influenza A virus was detected most frequently in December and January (30.3%, 12%, respectively) without infection of SARS-CoV-2. Conclusions: Some cases diagnosed with influenza before routine nucleic acid testing of SARS-CoV-2 were attributed to COVID-19. Co-infection between SARS-CoV-2 and other non-SARS-CoV-2 respiratory pathogens existed in the early stage of COVID-19 epidemic.

## 1. Introduction

The severe acute respiratory syndrome coronavirus 2 (SARS-CoV-2) was first discovered in Wuhan China in December 2019 and was later categorized as a pandemic in March 2020 [1,2,3]. The genomic characterization of SARS-CoV-2 shares 79.5% of the genetic sequence of the SARS-CoV-2 coronavirus that sparked the 2002–2003 epidemic [4,5]. SARS-CoV-2 received worldwide attention immediately after the outbreak and has become the most well-documented pandemic. There were 291 confirmed coronavirus disease 2019 (COVID-19) cases in China alone by 20 January 2020 [6], although this number was later reported to be even higher [7]. By the end of 2020, the number of confirmed cases was above 35 million [8].

The COVID-19 outbreak occurred during the 2019/2020 influenza season, which had not yet reached its peak in many countries. In spite of various predictions, the influenza season was similar to previous seasons in terms of incidence and mortality in many countries, including the United States [9], countries comprising the European Region [10], etc. Notably, an opposite trend was observed in Asia, where the number of reported influenza cases nationwide was one of the lowest in decades [11,12].

Given that symptoms of COVID-19 include fever, chills, cough, shortness of breath, fatigue, sore throat, and headache, which are similar to seasonal influenza [13], and that the outbreak occurred during the winter influenza season, in this study we describe the prevalence of commom respiratory pathogens and co-infection with SARS-CoV-2 in the early stage of COVID-19 epidemic.

For this study, frozen nucleic acid samples were obtained from patients admitted due to influenza-like symptoms to the Renmin Hospital of Wuhan University and the Shanxi Provincial People’s Hospital from 1 November 2019 to 20 January 2020 (date of clinical application of SARS-CoV-2 nucleic acid test). These nucleic acid samples were first sent to common respiratory pathogenic microorganisms detection and then SARS-CoV-2 detection.

## 2. Materials and Methods

### 2.1. Study Design

Patients that met the following four influenza-like illness criteria were included in the study: (1) acute onset of symptoms, (2) high fever, (3) respiratory infection symptoms, with or without (4) general symptoms, such as headache, diarrhea, chills, and myalgia. A total of 685 evaluated patients were hospitalized due to symptoms resembling influenza from 1 November 2019 to 20 January 2020 (616 patients from the Renmin Hospital of Wuhan University and 69 from the Shanxi Provincial People’s Hospital). Both hospitals are Tertiary Grade A Hospitals located in major cities in China.

### 2.2. Data Collection

Demographic, clinical, laboratory, radiological information, management, and outcome were obtained from patients’ medical records. Detection of common respiratory pathogenic microorganisms was based on the analysis of one-step Reverse Transcription-Polymerase Chain Reaction (RT-PCR). The rRT-PCR confirmed COVID-19 following the SARS-CoV-2 Pneumonia Prevention and Control Program (6th edition) issued by the National Health Commission of China.

Six hundred and eighty-five respiratory pathogenic microorganisms samples were collected from every flu-like patients. The samples were taken from nasopharyngeal swabs, deep sputum, and alveolar lavage fluid (613 nasopharyngeal swabs, 1 deep sputum, 2 alveolar lavage fluids from Wuhan, and 69 nasopharyngeal swabs from Taiyuan). They were then immersed in the sample preservation solution containing high concentrations of sodium isothiocyanate and protein denaturation reagents to inhibit the nuclease activity and stabilize the nucleic acids. After nucleic acid extraction, the remaining samples were stored at −80 °C.

### 2.3. Nucleic Acid Extraction and Preservation

By using Nucleic Acid Extraction or Purification Kit (Ningbo Health Gene Technologies Co., Ltd., Ningbo, China), cells and viruses were lysed, and nucleic acids were released. The nucleic acid extraction process was performed on the LabAssist-32 Nucleic Acid Extraction System (Shanghai Xingyao Medical Technology Development Co., Ltd., Shanghai, China). Nucleic acid samples were preserved at −80 °C.

### 2.4. Respiratory Pathogen Multiplex Detection

The 13 Respiratory Pathogen Multiplex Detection Kit (Ningbo Health Gene Technologies Co., Ltd., Ningbo, China) was employed to qualitatively detect 13 respiratory pathogens in nasopharyngeal swabs, deep sputum, and alveolar lavage fluid samples. The detected pathogens include influenza A virus (InfA), influenza A virus H1N1 (2009), seasonal H3N2 virus, influenza B virus (InfB), human adenovirus (ADV), human bocavirus (Boca), human rhinovirus (HRV), human parainfluenza virus (HPIV), Chlamydia (Ch), human metapneumovirus (HMPV), Mycoplasma pneumoniae (Mp), human coronavirus (HCOV), and human respiratory syncytial virus (HRSV). Thirteen sets of specific primers were employed, and one-step RT-PCR was performed in a single tube to amplify target fragments. The nucleic acid samples were amplified through a series of RT-PCR programs: first, pretreatment at 25 °C (5 min) for one cycle, reverse transcription at 50 °C (15 min) for one cycle, and predenaturation at 95 °C (2 min) for one cycle; secondly, denaturation at 94 °C(30 s), annealing at 65 °C (30 s), and extension at 72 °C (60 s) for six cycles, a step which was repeated until the temperature of annealing reaches 60 °C, and it reaches 1 °C touchdown for every six cycles; thirdly, denaturation at 94 °C (30 s), annealing at 60 °C (30 s), and extension at 72 °C (60 s) for 29 cycles; lastly, the products were extended at 72 °C (10 min) for one cycle and were kept at 4 °C for one cycle. Capillary electrophoresis was applied to separate the amplification products of different lengths by a 3500 Dx/3500xL Dx Genetic Analyzer.

### 2.5. SARS-CoV-2 Nucleic Acid Detection

By applying real-time fluorescence quantitative RT-PCR technology, Novel Coronavirus (2019-nCoV) Nucleic Acid Diagnostic Kit (Sansure Biotech, Changsha, China), which was employed for the conserved sequence of the double-target genes was used for the qualitative detection of SARS-CoV-2 ORF 1ab and specific conserved sequence of coding nucleocapsid protein N gene. FAM (ORF-1ab region) and ROX (N gene) channels were selected to test SARS-CoV-2 nucleic acid, and CY5 channel was tested as the internal control. The nucleic acid samples were amplified under the following conditions: reverse transcription at 50 °C (30 min) for one cycle, cDNA predenaturation at 95 °C (1 min) for one cycle, denaturation 95 °C (15 s) followed by annealing, extension, and fluorescence collection at 60 °C (30 s) for a total of 45 cycles, and device cooling at 25 °C (10 s) for one cycle. A sample was considered positive when there were typical S-shape amplification curves and Ct ≤ 40 detected for ORF1ab gene (FAM) and/or N gene (ROX). A sample was considered negative when internal control (CY5) was Ct ≤ 40, while ORF1ab gene and N gene were Ct > 40.

The rRT-PCR detection system used the positive internal control, which monitored the presence of PCR inhibitors in test specimens by detecting normal internal control signals to avoid a false-negative result.

### 2.6. Statistics Analyses

SPSS version 22.0 was employed for the analysis of the data. Continuous variables were described as the mean ± standard deviation (SD) if normally distributed; otherwise, they were expressed as median (interquartile range), mean, deviation, maximum, and minimum. The patients that did not reach 1 year of age were regarded as 1 year old. The chi-square test was used for comparisons among periods. A two-tailed *p*-value < 0.05 was considered statistically significant.

### 2.7. Ethics Approval Statement 

Participants involved provided written informed consent for the use of their clinical data in scientific research. The study was approved by the Ethics Committee of Shanxi Provincial People’s Hospital (Approval Number: No.8/2020) and the Clinical Research Ethics Committee of the Renmin Hospital of Wuhan University (Approval Number: WDRY2020-K061). We confirm that all experiments were performed in accordance with relevant named guidelines and regulations.

## 3. Results

### 3.1. Baseline Characteristics

Six hundred and sixteen patients’ samples were collected from Wuhan, including 326 men and 290 women. For men, the median age is 6 years (1–92 years). For women, the median age is 26.5 years (1–88 years). A total of 69 samples were recruited in Taiyuan, including 39 men and 30 women. The median age among men is 62 years (17–93 years) and among women is 56 years (56–86 years). Patients in Wuhan were younger than in Taiyuan (Mean ± SD age among males: 25.66 ± 28.53 years vs. 58.62 ± 19.47 years; mean ± SD among females: 29.94 ± 27.79 years vs. 51.03 ± 26.79 years). The majority of the patients were males in the two cities (52.9% in Wuhan and 56.5% in Taiyuan). The baseline characteristics of the enrolled patients are provided in Table 1.

### 3.2. Distribution of Non-SARS-CoV-2 Respiratory Pathogens

**Figure 1:**
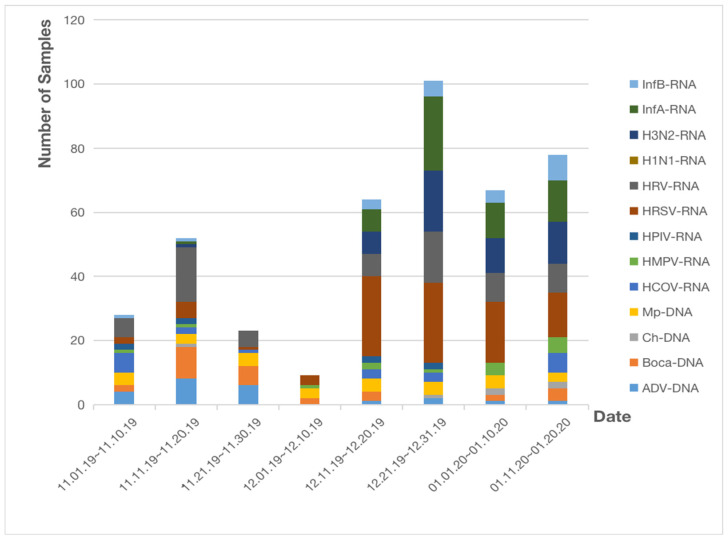
Infections of non-SARS-CoV-2 respiratory pathogens in Renmin Hospital of Wuhan University. Abbreviations: InfA: influenza A virus; InfB: influenza B virus; ADV: human adenovirus; Boca: human bocavirus; HRV: human rhinovirus; HPIV: human parainfluenza virus; Ch: Chlamydia; HMPV: human metapneumovirus; Mp: Mycoplasma pneumoniae; HCOV: human coronavirus; HRSV: human respiratory syncytial virus; H1N1: influenza A virus H1N1 (2009); H3N2: seasonal H3N2 virus; nCOV: novel coronavirus.

Six hundred and sixteen respiratory pathogenic microorganisms samples (including nasopharyngeal swabs, deep sputum and alveolar lavage fluid) collected from the Renmin Hospital of Wuhan University from 1 November 2019 to 20 January 2020 were included in the study. Infections of respiratory pathogenic microorganisms are shown in Figure 1.

During the first 10-day period (from 1 November to 10 November), HRV and HCOV emerged as the most frequent infectious pathogens (6/22 [27.3%]). During the second one (11 November to 20 November), HRV was the most frequent infectious pathogen (17/53 [32.1%]). During the third one (21 November to 30 November), ADV and Boca accounted for the biggest number of cases (6/14 [42.9%]). During the fourth one (from 1 December to 10 December), most of the infections were caused by HRSV and Mp (3/11 [27.3%]). During the fifth to the eighth 10-day period (from 11 December to 20 January), HRSV was the most commonly found pathogen (25/55 [45.5%], 25/77 [32.5%], 19/67 [28.4%], 14/317 [4.4%], respectively).

**Figure 2:**
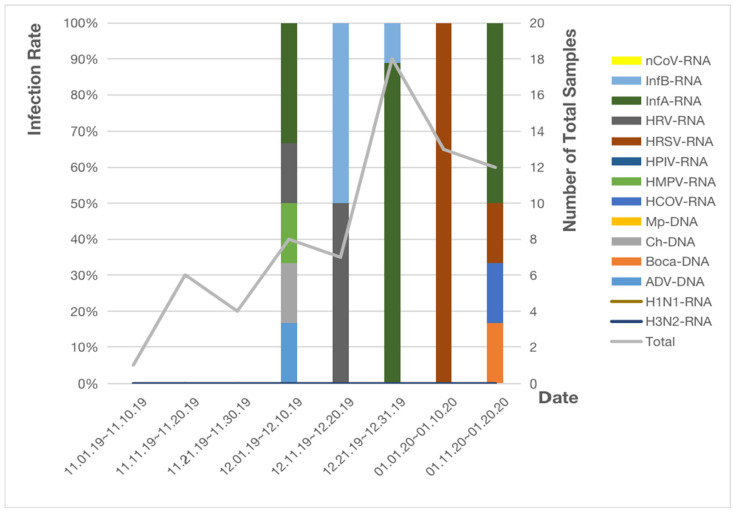
Infection of respiratory pathogenic microorganisms in Shanxi Provincial People’s Hospital.

Furthermore, 69 inspected samples from the Shanxi Provincial People’s Hospital were included in the analysis and detected pathogens included InfB, InfA, HRV, HRSV, HMPV, HCOV, Ch, Boca, and ADV. In the same way, during the first 10-day period to the third one (1 November to 30 November), none of 11 samples showed a positive result for infection with respiratory pathogenic microorganisms. During the fourth one (from 1 December to 10 December), InfA was the most infectious pathogen (2/8 [25%]). During the fifth one (from 11 December to 20 December), HRV and InfB were diagnosed in most cases (1/7 [14.3%]). During the sixth one (from 21 December to 31 December), most patients were infected with InfA (8/18 [44.4%]). During the last two 10-day periods (from 1 January to 20 January), infections with HRSV and InfA were the most frequent (1/13 [7.7%], 3/12 [25%], respectively) as is shown in Figure 2.

In total, the number of respiratory pathogenic microorganisms in the samples from Wuhan was as high as 103 (24.41%) in November, 174 (41.23%) in December, and 145 (34.36%) in January. The differences were statistically significant (*p* < 0.01). Whereas in Taiyuan, the number of respiratory pathogenic microorganisms was 17 (70.83%) in December and 7 (29.17%) in January. The differences are not statistically significant (*p* > 0.05), as is shown in Table 2.

### 3.3. Infection of SARS-CoV-2 and Characteristics of COVID-19 Patients

**Figure 3:**
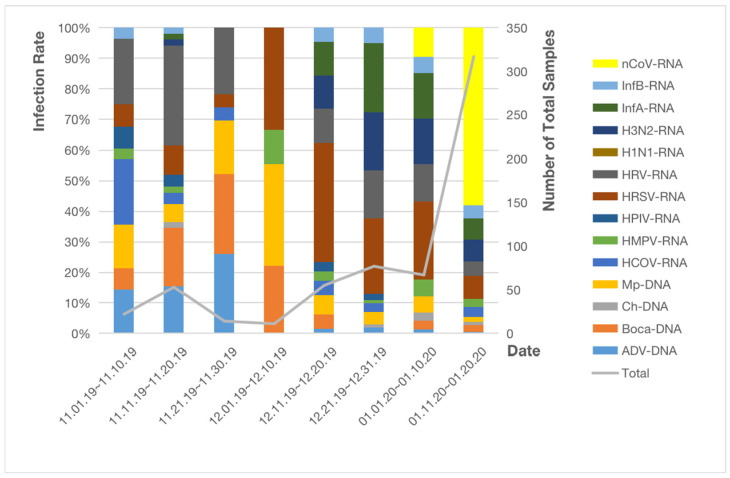
Infection rates of respiratory pathogenic microorganisms in Renmin Hospital of Wuhan University.

SARS-CoV-2 nucleic acid test was performed on 616 samples from Wuhan on 1 May 2020. The first samples positive for SARS-CoV-2 were collected from 1 January to 10 January (seven samples [10.45%]). From 11 January to 20 January the number of positive SARS-CoV-2 samples increased drastically (108 [34.07%]), outnumbering HRSV, the total number of samples reached 317 as shown in Figure 3. The difference is statistically significant between these two periods of time (*p* < 0.05) as is shown in Table 3. In general, 115 of 616 patients (18.7%) from Wuhan were diagnosed with COVID-19.

Seven positive samples were obtained from six patients, and they all had a fever as an initial symptom at the end of December, with cough (five [83.3%]), chills (two [33.3%]), emesis (one [16.7%]), and headache (one [16.7%]), as is shown in Table 4. Radiological examinations showed CT ground glass appearance was present in two (33.3%) of patients and bilateral chest CT consolidation was present in four (66.7%). Moreover, all six patients were treated by antibiotics. A steroid was used in two (33.3%) patients, the same as immunoglobulin and alveolar lavage treatment. The laboratory parameters of the six patients were analyzed and most of them were within normal range as is shown in Table 5. Some indicators had deviation from normal value maybe because of the sample size.

Five of six patients were hospitalized from 27 December 2019 to 2 January 2020, and discharged from 9 January to 16 January, without diagnosis of COVID-19. Only a 57-year-old woman, who was admitted to the hospital on 6 January and discharged on 4 February was diagnosed with SARS-CoV-2 infection. This is probably due to the clinical application of the SARS-CoV-2 nucleic acid test from 20 January.

The first patient in the study we could date back to was a 38-year-old man who developed fever, chills, and cough at the onset on 20 December 2019. He was admitted to the hospital with a fever on 27 December and discharged on 10 January. The earliest dates of admission and sample check-up were 27 December 2019, and 1 January 2020, respectively.

Moreover, HRSV and Mp were detected in two samples taken from children, respectively, they were also both positive for HMPV, whereas the rest of the four adults were not.

All the 69 samples from Taiyuan produced a negative result on the SARS-CoV-2 nucleic acid test.

## 4. Discussion

Our study sheds light on the early period of the SARS-CoV-2 outbreak. Our results indicate that co-infection between non-SARS-CoV-2 respiratory pathogens and SARS-CoV-2 existed at the beginning. We truthfully reflected the prevalence of respiratory pathogens through a retrospective study conducted in two major Chinese hospitals located in Wuhan and Taiyuan.

The earliest date of symptom onset in the study was 20 December 2019. We speculate that SARS-CoV-2 immediately broke out after it appeared in Wuhan without a long-term epidemic history. In contrast, 69 samples from Shanxi Provincial People’s Hospital were all infected with common respiratory pathogenic microorganisms. Taiyuan had not shown signs of the early epidemic of SARS-CoV-2 until 20 January 2020. Detection rates of SARS-CoV-2 and other respiratory pathogens reported in this study may be attributable to mask-wearing, handwashing, social distancing, etc. However, the effect of such preventive measures on the results of our study is dismal as human-to-human transmission of SARS-CoV-2 was not confirmed at that time, preventive measures just started being implemented, and they were taken seriously later by the public.

Among the six confirmed diagnoses of COVID-19 patients at the beginning of epidemic in Wuhan, four adult patients were diagnosed with SARS-CoV-2-positive infection with no other respiratory tract co-infections, while the other two underage patients presented with other microorganism superimposed infections. A six-year-old girl tested HRSV-positive and HMPV-positive during the alveolar lavage fluid test. The onset of fever and cough was recorded on 28 December 2019. Cephalosporins and imipenem antibiotics were administered along with IVIG and alveolar lavage treatment. On 10 January 2020, she was discharged. Her body temperature was 36.5 °C, though the patient had occasional coughs. The discharge diagnosis was severe pneumonia, respiratory syncytial virus infection and pleuritis. The other patient was a 4-year-old girl with combined HMPV and MP infections. On 29 December 2019, she was hospitalized due to cough, vomiting gastric content, and fever symptoms. Cephalosporins and azithromycin antibiotics were administered as infection management. On 9 January 2020, she was discharged with normal body temperature and occasional cough. The discharge diagnosis was asthmatic bronchial pneumonia, mycoplasma pneumonia infection, pneumococcal pneumonia, and Hemophilus influenza infection.

The present study adds to the existing knowledge of co-infection between SARS-CoV-2 and other viruses, particularly the influenza virus. Although early reports from China indicated that it was rare for SARS-CoV-2 to co-infect with other respiratory pathogens [14,15,16,17], recent studies and case reports of concurrent infections with influenza virus, human metapneumovirus (hMPV), and seasonal coronaviruses such as CoV-HKU-1 show that co-infection is not so rare and may influence mortality and morbidity [18,19,20]. Kim et al. reported that co-infections with SARS-CoV-2 were widespread in their patient population in Northern California. The patients often presented with most commonly parainfluenza virus, respiratory syncytial virus, rhinovirus/enterovirus, and non-SARS coronaviridae. Of 116 positive SARS-CoV-2 specimens, more than 20% also contained one or more additional respiratory pathogens. Moreover, 7.5% of their non-SARS-CoV-2 respiratory pathogen-positive specimens were SARS-CoV-2-positive [21]. However, studies of COVID-19 have not shown any difference in outcomes in patients with co-infection.

It has been found that co-infection occurred frequently among children infected with SARS-CoV-2. Research by Li Y et al. found that the rate of co-infection among COVID-19 patients was 33.3% in children, which was higher than that in adult [22]. COVID-19 children are more likely to co-infect with viral respiratory pathogens than their SARS-CoV-2-infected adult household contacts, according to Pigny F et al.’s study [23]. It has been reported that almost half of the SARS-CoV-2-infected children co-infected with other common respiratory pathogens [24]. A systematic review demonstrated that about 29% of pediatric cases had co-infection and children were more susceptible to co-infection than adults [25]. In our study, two children were co-infected among 115 SARS-CoV-2-positive samples from Wuhan, and other COVID-19 patients did not have co-infection with non-SARS-CoV-2 respiratory pathogenic microorganisms. Considerable attention should be paid to children patients.

Our study has certain limitations. Firstly, we emphasized the infection rate of SARS-CoV-2 and co-infection with other respiratory pathogens prior to the testing of SARS-CoV-2, so we did not collect clinical and laboratory results of all hospitalized patients. Only six COVID-19 patients during the first 10-day period were analyzed. Secondly, this is a retrospective study with the data from two hospitals of different provinces in China, thus the study sample size is relatively small. Nevertheless, to some extent, these data explained that the earliest date of COVID-19 onset was 20 December 2019. Thirdly, selection criteria of participants were based on clinical manifestations of influenza symptoms. SARS-CoV-2-positive individuals who had no symptoms initially were not included in the study.

## 5. Conclusions

In conclusion, this particular research sets out that some patients that were admitted to Chinese hospitals before 20 January 2020 (date of clinical application of SARS-CoV-2 nucleic acid testing) due to influenza-like symptoms were probably affected by SARS-CoV-2. Co-infection between non-SARS-CoV-2 respiratory pathogens and SARS-CoV-2 existed in the early stage of COVID-19 epidemic.

## Figures and Tables

**Table 1:** Baseline characteristics of 685 patients.

Cities	Age	P_25_	P_50_	P_75_	Mean	Deviation	Min	Max
Wuhan	Male (N = 326)	1.00	6.00	55.00	25.66	28.53	1	92
	Female (N = 290)	3.00	26.50	56.00	29.94	27.79	1	88
Taiyuan	Male (N = 39)	45.00	62.00	71.00	58.62	19.47	17	93
	Female (N = 30)	27.00	56.00	77.00	51.03	26.79	2	86

Footnote: P_25_: 25th Percentile; P_50_: Median; P_75_: 75th Percentile; Deviation: Standard Deviation.

**Table 2:** Non-SARS-CoV-2 respiratory pathogenic microorganisms.

City	Month	Infection ^a^	Total ^b^	Percentage (%)	X^2^	*p* Value
Wuhan	November	103	422	24.41	137	<0.01
	December	174		41.23		
	January	145		34.36		
Taiyuan	November	0	24	0	10.64	0.096
	December	17		70.83		
	January	7		29.17		

^a^ Infection: the number of nucleic acid samples positive for non-SARS-CoV-2 respiratory pathogenic microorganisms; ^b^ Total: the total number of infections samples in three months.

**Table 3:** Infection with SARS-CoV-2 in Wuhan.

City	Date	N ^a^	N ^b^	Percentage (%)	X^2^	*p*
Wuhan	1 January 2020–10 January 2020	67	7	10.45	14.71	<0.01
	11 January 2020–20 January 2020	317	108	34.07		

^a^ N: the total number of infected samples; ^b^ n: the number of samples infected with SARS-CoV-2.

**Table 4:** Characteristics of the initial six COVID-19 patients from 1 November 2019 to 20 January 2020.

Sex, n (%)	
Male	1 (16.7)
Female	5 (83.3)
Clinical symptoms, n (%)	
Fever	6 (100)
Cough	5 (83.3)
Emesis	1 (16.7)
Chills	2 (33.3)
Headache	1 (16.7)
Radiological features, n (%)	
CT ground glass appearance	2 (33.3)
Bilateral chest CT consolidation	4 (66.7)
Co-infection, n (%)	
HMPV	2 (33.3)
HRSV	1 (16.7)
Mp	1 (16.7)
Treatment, n (%)	
Antibiotics	6 (100)
Steroid	2 (33.3)
Immunoglobulin	2(33.3)
Alveolar lavage	2(33.3)
Admission Diagnosis, n (%)	
Fever of unknown	1 (16.7)
Pulmonary infection	3 (50)
Bronchitis	2 (33.3)
pneumonia	1 (16.7)
Discharge Diagnosis, n (%)	
Pneumonia	3(50)
Pulmonary infection	2(33.3)
Bronchitis	2(33.3)
COVID-19	1(16.7)

Footnote: CT: computed tomography.

**Table 5:** Laboratory indicators of the earliest six patients from 1 November 2019 to 20 January 2020.

Inspection Item	Mean ± SD	Reference Value
Erythrocyte count (×10^12^/L)	3.80 ± 0.82	3.50–5.00
Leukocyte count (×10^9^/L)	6.61 ± 5.52	4.00–10.00
Neutrophils count (×10^9^/L)	6.77 ± 5.28	2.00–7.00
Neutrophils%	74.38 ± 10.78	50.00–75.00
Monocytes count (×10^9^/L)	0.45 ± 0.31	0.20–1.00
Monocytes%	10.00 ± 5.40	3.00–8.00
Lymphocytes count (×10^9^/L)	1.20 ± 0.93	1.00–4.40
Lymphocytes%	20.18 ± 9.55	20.00–40.00
HGB (g/L)	119.33 ± 10.88	110.00–150.00
PLT (×10^9^/L)	356.25 ± 104.81	100.00–300.00
APTT (s)	35.05 ± 0.78	25.10–36.50
PT%	67.65 ± 8.84	80.00–160.00
FIB (g/L)	4.50 ± 0.34	2.38–4.98
AST (IU/L)	29.25 ± 22.72	0–40.00
ALB (g/L)	34.09 ± 2.99	38.00–60.00
HDL-C (mmol/L)	0.70 ± 0.16	0.80–1.80
Creatinine (μmol/L)	39.00 ± 12.36	53.00–106.00
Potassium (K) (mmol/L)	3.97 ± 1.33	3.50–5.50
Sodium (Na) (mmol/L)	139.67 ± 10.69	130.00–150.00
Calcium (Ca) (mmol/L)	1.95 ± 0.08	1.80–2.60
PH	7.46 ± 0.02	7.35–7.45
PCT (ng/mL)	0.09 ± 0.04	0–0.50
IgG (g/L)	11.37 ± 8.43	7.23–16.85

Footnote: SD: standard deviation; HGB: hemoglobin; PLT: Platelet; APTT: activated partial thromboplastin time; PT: prothrombin time; FIB: fibrinogen; AST: aspartate aminotransferase; ALB: albumin; HDL-C: high density lipoprotein cholesterol; PH: potential of hydrogen; PCT: procalcitonin; IgG: immunoglobulin G.

## Data Availability

The datasets generated or analyzed during the current study are not publicly available due to privacy concerns but are available from the corresponding author on reasonable request.
